# Plasma β_2_‐microglobulin and cerebrospinal fluid biomarkers of Alzheimer's disease pathology in cognitively intact older adults: the CABLE study

**DOI:** 10.1002/alz.087278

**Published:** 2025-01-09

**Authors:** Yi‐Ming Huang, Lan Tan, Jin‐Tai Yu

**Affiliations:** ^1^ QingDao University, QingDao Municipal Hospital, QingDao, ShanDong China; ^2^ Qingdao Municipal hospital, Qingdao university, Qingdao, Shandong China; ^3^ Huashan Hospital, Fudan University, Shanghai, Shanghai China

## Abstract

**Background:**

Previous studies have suggested a correlation between elevated levels of β_2_‐microglobulin (B2M) and cognitive impairment. However, the existing evidence is insufficient to establish a conclusive relationship. This study aims to analyse the link of plasma B2M to cerebrospinal fluid (CSF) Alzheimer's Disease (AD) biomarkers, and cognition.

**Method:**

To track the dynamics of plasma B2M in preclinical AD, 846 cognitively healthy individuals in the Chinese Alzheimer's Biomarker and LifestylE (CABLE) cohort were divided into four groups (suspected non‐AD pathology [SNAP], 2, 1, 0) according to NIA‐AA criteria. Multiple linear regression models were employed to examine plasma B2M’s relationship with cognitive and CSF AD biomarkers. Causal mediation analysis was conducted through 10,000 bootstrapped iterations to explore the mediating effect of AD pathology on cognition.

**Result:**

We found that the levels of plasma B2M were increased in stages 1 (P=0.0007) and 2 (P< 0.0001), in contrast to stage 0. In total participants, higher levels of B2M were associated with worse cognitive performance (P = 0.006 for MMSE; P= 0.012 for MoCA). Moreover, a higher level of B2M was associated with decreases in Aβ_1‐42_ (P<0.001) and Aβ_1‐42_/Aβ_1‐40_ (P=0.015) as well as increases in T‐tau/Aβ_1‐42_ (P<0.001) and P‐tau/Aβ_1‐42_ (P<0.001). Subgroup analysis found B2M correlated with Aβ_1‐42_ in non‐APOE ε4 individuals (P<0.001) but not in APOE ε4 carriers. Additionally, the link between B2M and cognition was partially mediated by Aβ pathology (percentage: 8.6% to 19.3%), whereas tau pathology did not mediate this effect.

**Conclusion:**

This study consolidated demonstrated the association of plasma B2M with CSF AD biomarkers as well as a possible important role of Aβ pathology in the association between B2M and cognitive impairment, particularly in cognitively normal individuals, particularly in cognitively normal individuals. The results indicated that B2M could be a potential biomarker for preclinical AD and might have varied functions throughout various stages of preclinical AD progression.

